# mTOR inhibitors in cancer therapy

**DOI:** 10.12688/f1000research.9207.1

**Published:** 2016-08-25

**Authors:** Jianling Xie, Xuemin Wang, Christopher G. Proud

**Affiliations:** 1 Nutrition and Metabolism, South Australian Health and Medical research Institute, Adelaide, SA, Australia; 2 School of Biological Sciences, University of Adelaide, Adelaide, SA, Australia

**Keywords:** mTOR, rapamycin, mTOR inhibitors, cancer therapy

## Abstract

The mammalian target of rapamycin, mTOR, plays key roles in cell growth and proliferation, acting at the catalytic subunit of two protein kinase complexes: mTOR complexes 1 and 2 (mTORC1/2). mTORC1 signaling is switched on by several oncogenic signaling pathways and is accordingly hyperactive in the majority of cancers. Inhibiting mTORC1 signaling has therefore attracted great attention as an anti-cancer therapy. However, progress in using inhibitors of mTOR signaling as therapeutic agents in oncology has been limited by a number of factors, including the fact that the classic mTOR inhibitor, rapamycin, inhibits only some of the effects of mTOR; the existence of several feedback loops; and the crucial importance of mTOR in normal physiology.

## A brief introduction to the mTOR pathway

mTOR (the mammalian or mechanistic target of rapamycin) is a protein kinase that forms two distinct types of multiprotein complex, termed mTOR complexes 1 and 2 (mTORC1 and mTORC2). Each plays key roles in cellular regulation ^1, 2^.

**Figure 1.**
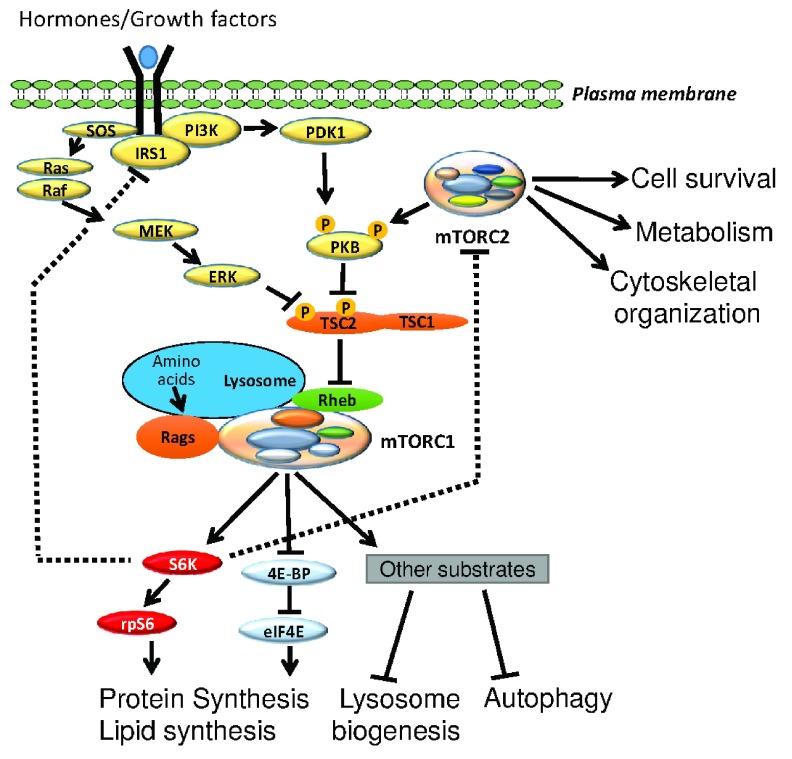
Schematic representation of signaling pathways involving the two mTOR complexes. Typically, hormones and growth factors activate mTOR complex 1 (mTORC1) through the SOS/Ras/Raf-MEK-ERK (MAPK) or the IRS1/PI3K-PDK1-PKB pathways or both. mTORC2 also contributes to the activation of PKB through the direct phosphorylation of its turn motif as well as its hydrophobic motif. These pathways impinge on the tuberous sclerosis complex (TSC), which serves as a GTPase activator protein for the small G-protein Rheb. Upon inhibitory phosphorylation evoked by upstream kinases such as PKB, the activity of TSC is suppressed, promoting the accumulation of GTP-bound Rheb, which in turn activates mTORC1 on the surface of lysosomes. Amino acids also activate mTORC1 by bringing the latter onto lysosomes via the Rag GTPases. S6K-rpS6 and 4EBP1-eIF4E are the best-characterized mTORC1 downstream targets and are responsible for controlling a variety of anabolic effects driven by mTORC1. Dashed lines indicate feedback mechanisms. mTOR, mammalian target of rapamycin; PI3K, phosphoinositide 3-kinase.

mTORC1 drives multiple anabolic pathways, including protein synthesis, ribosome production, lipogenesis, and nucleotide synthesis, all of which are important for cell and tissue growth ^1^. mTORC1 also suppresses a key catabolic process, autophagy ^3^, both by inhibiting its activation and by suppressing the production of lysosomes, the organelles in which autophagy occurs. mTORC1 phosphorylates proteins involved in all of these pathways, thereby altering their activities or subcellular localization ^3^. mTORC1 signaling is activated by several oncogenic pathways, including the Ras/Raf/MEK/ERK pathway and the phosphoinositide 3-kinase (PI3K)/AKT (PKB) pathway ( Figure 1), and by the intracellular availability of energy (ATP) and essential amino acids ^4, 5^. A key negative upstream regulator of mTORC1 is a protein complex which includes TSC1 and TSC2 ( Figure 1) ^6^. Loss of the gene for TSC1 or TSC2 leads to a condition termed tuberous sclerosis complex (TSC), which is characterized by benign tumors ^6^.

In particular, mTORC1 signaling positively regulates a key component of the cell’s protein synthesis machinery, eukaryotic initiation factor eIF4E, which mediates the recruitment of ribosomes to mRNAs for their translation ^7^. Enhanced expression of eIF4E can transform cells and is seen in various human cancers ^8^. Adequate levels of eIF4E are required for tumorigenesis ^8^. Its function is blocked by small phosphoproteins termed eIF4E-binding proteins (4E-BPs). They are phosphorylated by mTORC1 and this induces their release from eIF4E, thereby alleviating such inhibition ^7^. There are other links from mTORC1 to the activation of protein synthesis and to the production of ribosomes ^9, 10^.

mTORC2 has distinct substrates from mTORC1 ^11^, which include AKT (PKB) ^12^, a protein kinase that is involved in anabolic signaling (for example, in the activation of mTORC1 ^13– 15^ and in cell survival ^16^), and SGK1, whose function is rather less well understood. Owing to the lack of a specific inhibitor, much less is known about the control of mTORC2 than that of mTORC1. It may be linked to the PI3K pathway ^17^, which is frequently dysregulated in cancer, for example, by loss of the tumor suppressor protein PTEN (phosphatase and tensin homolog).

Given the many oncogenic pathways—and oncogenes or tumor suppressors—linked to mTOR signaling, it is estimated that mTORC1 function is hyperactivated in up to 70% of all human tumors ^18^. Equivalent information is not available for mTORC2, but its links to PI3K/PTEN suggest that it is also activated in tumor cells. This has stimulated a very high level of interest in targeting mTOR for cancer therapy; a search in PubMed for ‘mTOR inhibitors cancer therapy + review’ returns more than 1,000 hits.

These features have led to a very high level of interest—in academic labs and in the pharmaceutical industry—in targeting mTOR signaling as a potential therapeutic avenue for anti-cancer therapy.

## Rapamycin and the first generations of mTOR inhibitors

**Figure 2.**
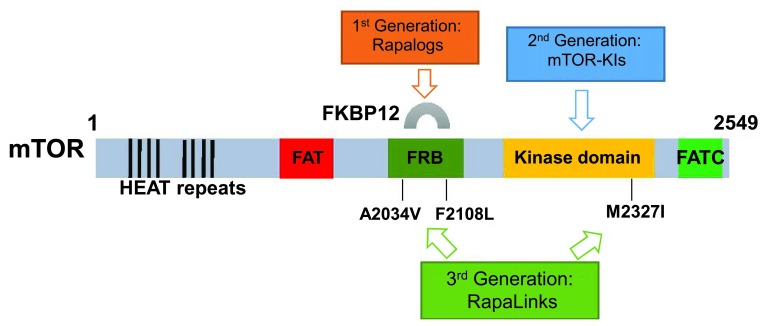
Domains of the mTOR protein and three generations of mTOR inhibitors. mTOR is composed of 2,549 amino acids which can be divided into several structural domains, including HEAT (for anti-parallel α-helices found in Huntingtin, elongation factor 3, PP2 A and TOR1) repeats and FAT (for FRAP, ATM, TRAP), FRB, kinase, and FATC (for *C*-terminal FAT) domains. The HEAT repeats, located close to the *N*-terminus of mTOR, are required for mTOR multimerization. The FRB—FK506 binding protein 12 (FKBP12)–rapamycin binding—domain, as its name implies, is the binding site of mTOR to FKBP12 and rapamycin. FAT, kinase, and FATC domains are conserved within the phosphatidylinositol 3-kinase-related kinases (PIKKs) and are essential for maintaining the activity of PIKKs. The first-generation mTOR inhibitors, including rapamycin itself, bind to FKBP12, which in turn interacts with the FRB domain of mTOR to inhibit mTOR activity. The second-generation mTOR inhibitors are ATP-competitive mTOR inhibitors which act as ATP analogues and compete with ATP for the binding to the kinase domain of mTOR. The newly developed third generation of mTOR inhibitors can potentially overcome the drug resistance of cancer cells bearing mTOR FRB/kinase domain mutation; that is, FRB domain mutations (mTOR ^A2034V^ and mTOR ^F2108L^) confer resistance to rapalogs (first generation), and a kinase domain mutation (mTOR ^M2327I^) renders resistance to mTOR-KIs (second generation). mTOR, mammalian target of rapamycin.

The best-known inhibitor of mTOR is rapamycin, from which mTOR’s name derives. Rapamycin was originally applied as an immunosuppressant, blocking T-cell activation, and has been in use since around 2000 to prevent kidney graft rejection. However, rapamycin does not directly inhibit the catalytic (kinase) activity of mTOR; instead it binds, together with a small protein, an immunophilin termed FKBP12, specifically to mTORC1, but not mTORC2, to a domain adjacent to the kinase active site ( Figure 2). As a consequence, it inhibits only some of the functions of mTORC1. The data suggest that its effect on mTORC1 activity affects mainly weaker mTORC1 substrates, such as the protein kinase termed ribosomal protein rpS6 kinase, whereas it has only a limited, if any, effect on other, better substrates such as the eIF4E-binding protein 4E-BP1 ^19^. The extent of its effect on this latter substrate appears to vary between cell types. In contrast to a previous report by Yip *et al*. ^20^, which showed that rapamycin treatment results in destabilization of the hollow lozenge-shaped mTORC1 dimer, a recent high-resolution cryo-electron microscopy study from Aylett *et al*. ^21^ showed that the binding of rapamycin-FKBP12 to mTOR does not destabilize the mTORC1 dimer but rather reduces the access to the active site cleft from a width of 20 to 10 Å, implying that the FKBP12-rapamycin binding (FRB) domain acts as a gatekeeper of the active substrate binding site (discussed in 22). This may well explain why rapamycin differentially affects the phosphorylation of ‘stronger’ versus ‘weaker’ substrates.

4E-BP1 is the substrate through which mTORC1 controls cell proliferation ^23^, so the resistance of its phosphorylation to rapamycin likely contributes to the poor efficiency of rapamycin as an anti-hyperplastic agent. Another confounding factor is that, by impairing mTORC1, rapamycin can promote growth factor signaling via various feedback loops which, for example, promote activation of the oncogenic PI3K/Akt pathway ^24, 25^. In addition, rapamycin is generally not cytotoxic, acting instead as a cytostatic agent. It can cause the upregulation of the pro-oncogenic protein eIF4E and promote other tumorigenic events (reviewed in 26). Tumors expressing high levels of eIF4E are likely to be less sensitive to mTOR inhibitors since levels of eIF4E may exceed those of the mTORC1-regulated inhibitor protein 4E-BP1. Inhibition of mTORC1 will also activate its downstream effector, eukaryotic elongation factor 2 kinase (eEF2K), which can promote cell survival ^27^.

As noted, it is very common that cellular signaling pathways involving the mTOR complexes are abnormally upregulated in cancer. Although rapamycin is a highly selective inhibitor against mTOR, it does not completely inhibit all of the activities of mTORC1 ^28^ and will inhibit mTORC2 in only some types of cells upon prolonged treatment ^29^. Although rapamycin does not interact with mTORC2, it can affect mTORC2 indirectly. By binding to mTOR as a complex with FKBP12, it prevents mTOR from associating with the mTORC2-specific partner protein Rictor, therefore causing a gradual decline in mTORC2 levels ^29^. The rate at which this occurs will depend on the rate of turnover of mTORC2 under given conditions but can occur within 1 or 2 days of treatment of cells with rapamycin and may account for some of the longer-term effects of rapamycin ^29^. Also, S6K can phosphorylate Rictor and thereby impair mTORC2 function, an effect that should be reversed by rapamycin ^30, 31^. These opposing effects are important considerations when interpreting—or trying to predict—the consequences of rapamycin treatment.

**Figure 3.**
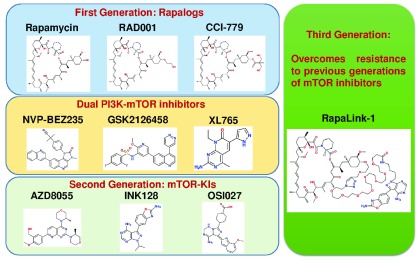
Selected examples of three generations of mTOR inhibitors and dual PI3K/mTOR inhibitors. Chemical structures were drawn by using the website www.emolecules.com. mTOR, mammalian target of rapamycin; PI3K, phosphoinositide 3-kinase.

The pharmacological properties of rapamycin itself are not ideal, leading to the development and application of rapamycin analogs (rapalogs) with superior characteristics. Several such compounds have been developed and evaluated for their efficacy in treating diseases, including cancers ( Figure 2 and Figure 3 and Table 1). These are semi-synthetic rapamycin analogues which have been typically derivatized at the C-43 position on the cyclohexane outside the macrolide ring in order to improve aqueous solubility and suit oral administration. This also provides a more advantageous intellectual property position than for rapamycin itself. For example, RAD001 (everolimus) ^32, 33^, developed by Novartis as an immunosuppressive and anti-cancer drug ( Table 1), is a hydroxyethyl ether-derivative. CCI-779 (temsirolimus; Wyeth-Ayerst/Pfizer) and AP23573 (ridaforolimus or deforolimus; Merck/Ariad) also belong to this category.

**Table 1.** Examples of the three generations of rapalogs/dual mTOR/PI3K inhibitors and their effects on human diseases.

Generation	Compound name	Approved year or current phase	Developer	Examples of indications in completed clinical trials	Reference
1st	Rapamycin (sirolimus)	1999	Wyeth-Ayerst	Acute renal allograft rejection/ restenosis	77– 80
1st	RAD001 (everolimus)	2003–2011	Novartis	Allograft rejection/advanced kidney cancer/ tuberous sclerosis/advanced RCC/pNET/ neurofibromatosis	32, 33
1st	CCI-779 (temsirolimus)	2007–2008	Wyeth-Ayerst/Pfizer	Advanced RCC/mantle cell lymphoma	81
DI	NVP-BEZ235 (dactolisib)	Phase I/II (22)	Novartis	MBC/pNET	35
DI	GSK2126458	Phase I/II (3)	GlaxoSmithKline	Advanced solid tumors, lymphoma	38
DI	XL765	Phase I/II (5)	Sanofi-Aventis, Exelixis	Glioblastoma multiforme/NSCLC/ MBC	39
2nd	AZD8055	Phase I/II (5)	AstraZeneca	Advanced solid tumors/glioma/HCC	50
2nd	INK128/MLN0128	Phase I/II (25)	Intellikine	Advanced solid tumors/multiple myeloma/Waldenstrom macroglobulinemia	44
2nd	OSI027	Phase I/II (1)	OSI Pharmaceuticals	Advanced solid tumors/lymphoma	82
3rd	RapaLinks	Developed in 2016	Rodrik-Outmezguine *et al*.	Tested in rapamycin- and AZD8055- resistant cell lines and mouse xenografts	74

For “current phase”, the number within the parentheses indicates the number of clinical trials currently being carried out or already withdrawn, completed, or terminated according to ClinicalTrials.gov. DI, dual mammalian target of rapamycin/phosphoinositide 3-kinase inhibitor; HCC, hepatocellular carcinoma; MBC, metastatic breast cancer; mTOR, mammalian target of rapamycin; NSCLC, non-small cell lung cancer; PI3K, phosphoinositide 3-kinase; pNET, pancreatic neuroendocrine tumor; RCC, renal cell carcinoma.

Despite the strong evidence that mTORC1 and mTORC2 control events that are important for cell growth and survival, which are processes of key importance in cancer cells, progress in successfully applying rapamycin and rapalogs as anti-cancer agents has been limited. Temsirolimus was approved by the US Food and Drug Administration for advanced renal cell carcinoma in 2007 and since then everolimus has been passed for use in certain other cancers, including neuroendocrine tumors and, as a combination therapy, HER2-positive breast cancer, as well as for certain TSC-related tumors ( Table 1). However, this relatively modest list contrasts to the immense amount of research effort devoted to studying mTORC1 signaling in tumors or cancer cells.

## Dual PI3K/mTOR inhibitors

The FAT, FATC, and kinase domain of mTOR are widely conserved in a group of protein kinases which display prominent structural similarities to PIKK (PI3K-related kinases) ( Figure 2 and Table 1). As a result, it has been discovered that several PI3K inhibitors (including derivatives of the classic LY294002 and Wortmannin) developed during drug discovery projects can also effectively suppress the activation of both mTOR complexes. These are therefore classified as dual PI3K/mTOR inhibitors. As potential anti-cancer agents, they represent superior benefits in comparison with the first class of mTOR inhibitors because they simultaneously inhibit both PI3K and mTOR, two crucial signaling hubs that promote cancer cell growth. The first inhibitors that came out from this group of compounds were PI103 and its derivatives, named PI450 and PI620, which show further improvements to the pharmacokinetic properties of the parent molecule ^34^. Yet arguably the most successful example in clinical trials from this class of inhibitors is the imidazoquinoline derivative NVP-BEZ235 ^35^ developed by Novartis. Not only does the dual mTOR/PI3K inhibitor NVP-BEZ235 exert potent anti-tumor activity *in vivo* but its effect can be further enhanced by the combination with inhibitors against other mitogenic pathways, such as the MEK/ERK inhibitors ^36, 37^. Other examples from this class of inhibitors include GSK2126458 from GlaxoSmithKline ^38^, XL765 from Sanofi-Aventis and Exelixis ^39^, and SF1126 from Semafore ^40^.

## Second-generation mTOR inhibitors

Given the inability of rapamycin to affect all functions of mTORC1 and its inefficacy in anti-cancer therapy, several academic and pharmaceutical laboratories have developed compounds that inhibit the catalytic activity of mTOR itself. This means they can potentially inhibit all phosphorylation events catalyzed by mTORC1 but will also affect mTORC2. This finally gave rise to a second generation of mTOR inhibitors which are designed to act as ATP-competitive agents to mTOR. These inhibitors exhibit a much lower half-maximal inhibitory concentration (IC _50_) against mTOR activity than PI3K. The first such compound is the mTOR inhibitor PP242 ^41^. As a classic indication of complete mTORC1 inhibition, the phosphorylation of the rapamycin-resistant sites in 4E-BP1 (Thr37/Thr46) is effectively blocked by PP242 ^42^. PP242 also shows effectiveness against rapamycin-resistant PKB-driven tumorigenesis ^43^. INK128 (later renamed MLN0128) is a PP242 derivative developed by Intellikine ^44^ and has been, or is being, tested in 25 clinical trials, according to ClinicalTrials.gov ( Table 1).

Within the same category, Torin 1 ^45^ and its sister compound Torin 2 ^46^ were synthesized from quinolone 1 by Nathanael Gray’s lab and developed by AstraZeneca. These compounds exhibit an IC _50_ against mTOR of less than 10 nM (3 nM as for Torin 1) *in vitro*. Torin 2 not only exhibits approximately 100-fold selectivity relative to PI3K (IC _50_ of approximately equal to 200 nM) and 100-fold selectivity over other kinases tested but also possesses enhanced bioavailability and stability. Ku-0063794 and Ku-0068650 ^47, 48^, developed by KuDOS Pharmaceuticals (now part of AstraZeneca), are also examples of early ATP-competitive mTOR inhibitors which exhibit great anti-proliferative potential against cancer cells *in vitro*. Wyeth-Ayerst (now part of Pfizer) developed a series of dual mTORC1/2 inhibitors via high-throughput screening based on the parent compound WAY-001 and subsequently named them WAY-600, WYE-687, and WYE-354 ^49^. These compounds also possess anti-proliferative effects on cancer cells and have similar IC _50_ values toward mTOR as the Torin and Ku compounds and have reasonable selectivity for mTOR as compared to the PI3Ks (approximately 100-fold). Moreover, AZD8055 and AZD2014 ^50, 51^ are two orally bioavailable compounds derived from the Ku compounds. They were developed by researchers from KuDOS Pharmaceuticals and later AstraZeneca. The effectiveness of these compounds in the inhibition of cancer cell growth has been tested in several cancer cell lines where they show an anti-proliferative IC _50_ dose range of 20 to 50 nM ^50^. AZD2014 is currently being tested in combination with other inhibitors, including ibrutinib (which blocks B-cell receptor signaling), AZD6244 (a MEK inhibitor), paclitaxel (targets tubulin), and fulvestrant (estrogen receptor degrader), in phase I/II clinical trials against breast cancer, lung cancer, and lymphoma.

## Side effects of previous mTOR inhibitors and the birth of a new generation

Despite the high efficiency in inhibiting the activity of both mTOR complexes, ATP-competitive mTOR inhibitors are still quite ineffective in our battle against cancer, potentially for several reasons. Firstly, the inhibition of mTORCs triggers a number of feedback loops toward upstream signaling pathways, activation of which may promote cancer cell survival and metastasis ^31^. These pathways have been discussed in some detail by Li *et al*. ^26^.

Secondly, mTOR signaling is essential for normal cell viability and its inhibition can be unavoidably detrimental to healthy tissues. For instance, sirolimus and tacrolimus were given as immunosuppressant drugs during pancreatic islet transplantations ^52^, yet a follow-up study 5 years later has demonstrated that only approximately 10% of the recipients remained insulin independent ^53^, likely owing to the fact that mTOR inhibitors would induce pancreatic β-cell death ^54, 55^ as a result of the inhibition of mTORC2 ^56^. Important to note is that second-generation mTOR inhibitors, such as Torin 1, are actually more toxic to islet cells than rapamycin itself ^56^, potentially because of its rapid and complete suppressive action against both mTOR complexes. Furthermore, mTORC1 is a well-characterized as inhibiting of autophagy ^3^, and the induction of autophagy caused by mTOR inhibition may promote cancer cell survival. Indeed, AZD8055 is shown to activate autophagic flux in a variety of cancer cells ^50, 57, 58^ and the inhibition of autophagy was able to reverse the paradoxical cytoprotective effect of AZD8055 on colon carcinoma cells ^57^. Also, mTOR is a master positive regulator of mRNA translation, which is carried out by versatile high energy-consuming molecular machineries within the cell, and because energy saving is crucial for cancer cell survival as a result of the Warburg effect ^59^, mTOR inhibition may actually protect cancer cells from death by conserving essential energy to maintain cell viability.

One of the key targets for control by mTORC1, eIF4E, is often expressed at high levels in tumors ^8^; if its levels exceed those of its mTORC1-regulated inhibitors, 4E-BPs, then inhibition of mTORC1 will not be effective in restricting eIF4E function ^60^. Alternative ways of impairing eIF4E function may be effective in such settings; possibilities include the use of anti-sense RNAs against its mRNA ^61^, and blocking its binding to its partner eIF4G ^62^.

Given, on one hand, the likely importance of mTORC1 signaling in cancer and, on the other hand, the challenges of targeting mTORC1 in a safe and effective way, an alternative strategy is to block the relevant, key events downstream of mTORC1. Kinases that phosphorylate rpS6 are phosphorylated and activated by mTORC1. There are two genes in mammals, often termed *S6K1* and *S6K2*, each of which gives rise to two protein isoforms ^63^. These enzymes should not be confused with the RSKs, which are named after their ability to phosphorylate rpS6 but are regulated by the oncogenic Ras/Raf/MEK/ERK pathway, not by mTORC1 ^64^. Although the functional significance of the phosphorylation of rpS6 is unclear, S6K1 in particular regulates cell growth (size) ^65, 66^. This almost certainly reflects a role for S6K1 in controlling ribosome biogenesis ^67, 68^, a key process for cell growth control, although additional events may also be involved.

Another route that is being actively explored is to inhibit RNA polymerase I, which makes the main ribosomal RNAs and is switched on by mTORC1 signaling (reviewed in 69). Ribosome production is crucial in cell growth and proliferation and so inhibiting this pathway holds the potential for inhibiting tumor growth. Bywater *et al*. have developed an inhibitor of Pol I, CX-5461 ^70^, which, when used in combination with everolimus, extended the survival of mice with myc-driven lymphoma ^71^.

A final issue compromising the efficacy of mTOR inhibitors is that a wide range ^72– 76^ of clinically relevant mutations in mTOR can increase the catalytic activity of mTOR and thus both mTORC1 and mTORC2, thereby reducing the effectiveness of such compounds toward the first two generations of mTOR inhibitors and dual PI3K/mTOR inhibitors in cancer cells ^72– 76^. This mainly reflects increases in mTOR kinase activity caused by such mutations rather than interference with drug binding as a result of active site mutations ^74^; since catalytic activity is higher, a dose of inhibitor that is effective against wild-type mTOR will still leave appreciable catalytic activity of the mutant, hyperactive mTOR kinase.

To try to tackle the last of these issues, Rodrik-Outmezguine *et al*. ^74^ generated rapamycin-resistant breast cancer cell lines (MCF-7 and MDA-MB-468) carrying two mTOR FRB domain mutations ( *MTOR* ^A2034V^ and *MTOR* ^F2108L^) as well as an AZD8055-resistant colony bearing a hyperactive kinase domain mutation ( *MTOR* ^M2327I^). Careful analysis of the molecular model of mTOR revealed a juxtaposition of the rapamycin and AZD8055 binding sites, prompting the authors to create a powerful bivalent mTOR inhibitor, named RapaLink. This contains both rapamycin and an mTOR kinase inhibitor within the same molecule, connected by a cunningly designed non-perturbing, strain-free cross-linker with the optimum length, which allows the compound to interact with the FRB domain of mTOR through binding to FKBP12 and also to reach the kinase domain of mTOR so that it can also act as an ATP-competitive inhibitor at the same time ^74^ ( Figure 2 and Figure 3). Indeed, 3 to 10 nM of either RapaLink-1 or -2 is sufficient to inhibit both mTORC1 and 2 in these mutant cells, as demonstrated by the phosphorylation status of their respective downstream targets, whereas rapamycin and INK128 were unable to effectively block the activities of mTORCs at concentrations of as high as 100 nM ^74^. Mouse xenografts of MCF-7 cells bearing these mutations are also more sensitive to RapaLink-1 in comparison with rapamycin and AZD8055 ^74^. This landmark study has given birth to a new generation of mTOR inhibitors.

## Concluding comments

It is not surprising, given its importance in normal physiology and in various disease states, that so much attention has been devoted to understanding mTOR signaling pathways and to developing agents that interfere with signaling through mTOR. Despite this effort, the utility of such inhibitors in oncology still appears to be limited for reasons described above. One potential way forward is to develop ways of inhibiting those steps downstream of mTOR, especially mTORC1, that play critical roles in oncogenesis and tumor progression.
